# Coxsackievirus Cloverleaf RNA Containing a 5′ Triphosphate Triggers an Antiviral Response via RIG-I Activation

**DOI:** 10.1371/journal.pone.0095927

**Published:** 2014-04-23

**Authors:** Qian Feng, Martijn A. Langereis, David Olagnier, Cindy Chiang, Roel van de Winkel, Peter van Essen, Jan Zoll, John Hiscott, Frank J. M. van Kuppeveld

**Affiliations:** 1 Virology Division, Department of Infectious Diseases and Immunology, Faculty of Veterinary Medicine, University of Utrecht, Utrecht, The Netherlands; 2 Division of Infectious Diseases, Vaccine and Gene Therapy Institute of Florida, Port Saint Lucie, Florida, United States of America; 3 Department of Medical Microbiology, Radboud University Nijmegen Medical Centre, Nijmegen, The Netherlands; Kantonal Hospital St. Gallen, Switzerland

## Abstract

Upon viral infections, pattern recognition receptors (PRRs) recognize pathogen-associated molecular patterns (PAMPs) and stimulate an antiviral state associated with the production of type I interferons (IFNs) and inflammatory markers. Type I IFNs play crucial roles in innate antiviral responses by inducing expression of interferon-stimulated genes and by activating components of the adaptive immune system. Although pegylated IFNs have been used to treat hepatitis B and C virus infections for decades, they exert substantial side effects that limit their use. Current efforts are directed toward the use of PRR agonists as an alternative approach to elicit host antiviral responses in a manner similar to that achieved in a natural infection. RIG-I is a cytosolic PRR that recognizes 5′ triphosphate (5′ppp)-containing RNA ligands. Due to its ubiquitous expression profile, induction of the RIG-I pathway provides a promising platform for the development of novel antiviral agents and vaccine adjuvants. In this study, we investigated whether structured RNA elements in the genome of coxsackievirus B3 (CVB3), a picornavirus that is recognized by MDA5 during infection, could activate RIG-I when supplied with 5′ppp. We show here that a 5′ppp-containing cloverleaf (CL) RNA structure is a potent RIG-I inducer that elicits an extensive antiviral response that includes induction of classical interferon-stimulated genes, as well as type III IFNs and proinflammatory cytokines and chemokines. In addition, we show that prophylactic treatment with CVB3 CL provides protection against various viral infections including dengue virus, vesicular stomatitis virus and enterovirus 71, demonstrating the antiviral efficacy of this RNA ligand.

## Introduction

Pathogen recognition is a key step in the initiation of the host antiviral innate immune response. Specialized pattern recognition receptors (PRRs) such as Toll-like receptors (TLRs) and RIG-I-like receptors (RLRs) detect evolutionarily conserved structures known as pathogen-associated molecular patterns (PAMPs) and initiate an antiviral response. TLRs detect various PAMPs commonly found in both bacteria and viruses, such as lipopolysaccharides (LPS), and non-methylated CpG-containing DNAs (reviewed in [1]). Although TLRs are highly important in pathogen recognition, these receptors are mostly found at the cell surface and/or endosomes in specialized cell types (e.g. dendritic cells and macrophages). RLRs are cytosolic sensors present in virtually all nucleated cells, and thereby sense intracellular pathogens at the site of infection [2]. RIG-I, a well-studied RLR, specializes in recognizing dsRNAs containing 5′ triphosphate (5′ppp) groups (reviewed in [3]). Upon ligand engagement, RIG-I interacts with the mitochondrial adaptor molecule MAVS (also called IPS-1), which, in turn, interacts with TANK-binding kinase 1 (TBK1) and IκB kinase (IKK) complexes, leading to the activation of IRF3 and NF-kB, respectively. These transcription factors then activate transcription of type I interferons (IFNs) and many pro-inflammatory cytokine genes [2].

Type I IFNs play essential roles in combating viral infections by inducing expression of hundreds of interferon-stimulated genes (ISGs), which together establish an antiviral state in cells [2]. In addition, type I IFNs can activate components of the adaptive immune system such as natural killer cells and dendritic cells, and thereby orchestrate the emergence of the adaptive antiviral response [4], [5]. The efficacy of type I IFNs against viral infections has not only be shown at the molecular level in the laboratory but has also been demonstrated in the clinic, where pegylated IFN-α has been used extensively as a therapeutic to treat infections of hepatitis B and C viruses [6], [7]. However, clinical efficacy is accompanied by severe side effects such as nausea, hematological toxicity and depression [8], symptoms that lower the quality of life of recipients and also hamper adherence.

Recently, major efforts have been directed to the use of PRR agonists as antiviral agents. A TLR9 agonist, CpG oligodeoxynucleotides, was shown to prevent coxsackievirus B3 (CVB3)-induced myocarditis [9]. Pretreatment with polyinosinic:polycytidylic acid (poly(I:C)), a dsRNA mimic and ligand of TLR3 and RLRs, suppressed Hendra virus infection [10]. Recently, RNA ligands of RIG-I have also been reported to be highly immune stimulatory: a short, 5′ppp-containing RNA derived from the 3′ UTR of foot-and-mouth disease virus was shown to be an efficient type I IFNs inducer that protected against challenge viral infections [11]; two 5′ppp-containing RNA hairpins, both of which effectively activated the RIG-I signaling pathway, were shown to elicit protective antiviral immunity [12], [13]. Employing a systems analysis approach, Goulet et al. demonstrated that a VSV hairpin induced an extensive, broad spectrum of RIG-I responsive genes compared to the profile of interferon stimulated genes (ISGs) induced by recombinant type I IFNs, thus providing a more complete and balanced immune activation [13]. Together, these results indicate that RIG-I agonist may be promising candidates as antiviral agents.

From *in vitro* studies, it is known that RIG-I activation requires short double-stranded RNAs containing an intact 5′ppp group [14], [15]. A number of viral RNA species have been reported to activate RIG-I, including the well-known panhandle RNA structures of many negative-strand RNA viruses [2]. In addition, 5′ppp-containing *in vitro* transcripts of the Epstein–Barr virus (EBV)-encoded small RNAs (EBERs) [16], the Leader transcript of measles virus [17], and the polyU/UC tract from the 3′ UTR of hepatitis C virus (HCV) genome [18] have also been shown to activate RIG-I. Remarkably, the secondary structures of these RNA elements vary considerably – the panhandle RNAs are hairpin-shaped, containing a short double-stranded stem and a relatively large single-stranded loop [19], whereas the EBERs form more complex structures involving multiple stems with bulges and loops [20]; and the polyU/UC tract of HCV is, in fact, believed to be a linear stretch of RNA [18]. These data indicate that the recognition of RNA structures by RIG-I is perhaps more complex than currently understood.

Picornaviruses do not produce 5′ppp-containing RNA species during infection, and are known to activate MDA5, but not RIG-I, through its double-stranded replication intermediate [21]. However, picornavirus genomic RNA contains several well-studied, stable RNA structures that could potentially serve as potent RIG-I ligands when artificially provided with 5′ ppp moiety. In this study, we investigated whether any of these structured RNA elements, when provided with 5′ppp by *in vitro* transcription, would activate RIG-I signaling and the antiviral response. We demonstrate that a coxsackievirus B3 (CVB3) cloverleaf containing a 5′ppp moiety stimulated the RIG-I pathway, but other RNA stem-loop structures of similar lengths only poorly activated RIG-I, demonstrating the complexity and specificity of ligand recognition by this receptor. These results contribute to the identification and characterization of RIG-I ligands as potential antiviral agents.

## Results

### T7 transcripts of the first 1000 nt of CVB3 sequence activates RIG-I

**Figure 1:**
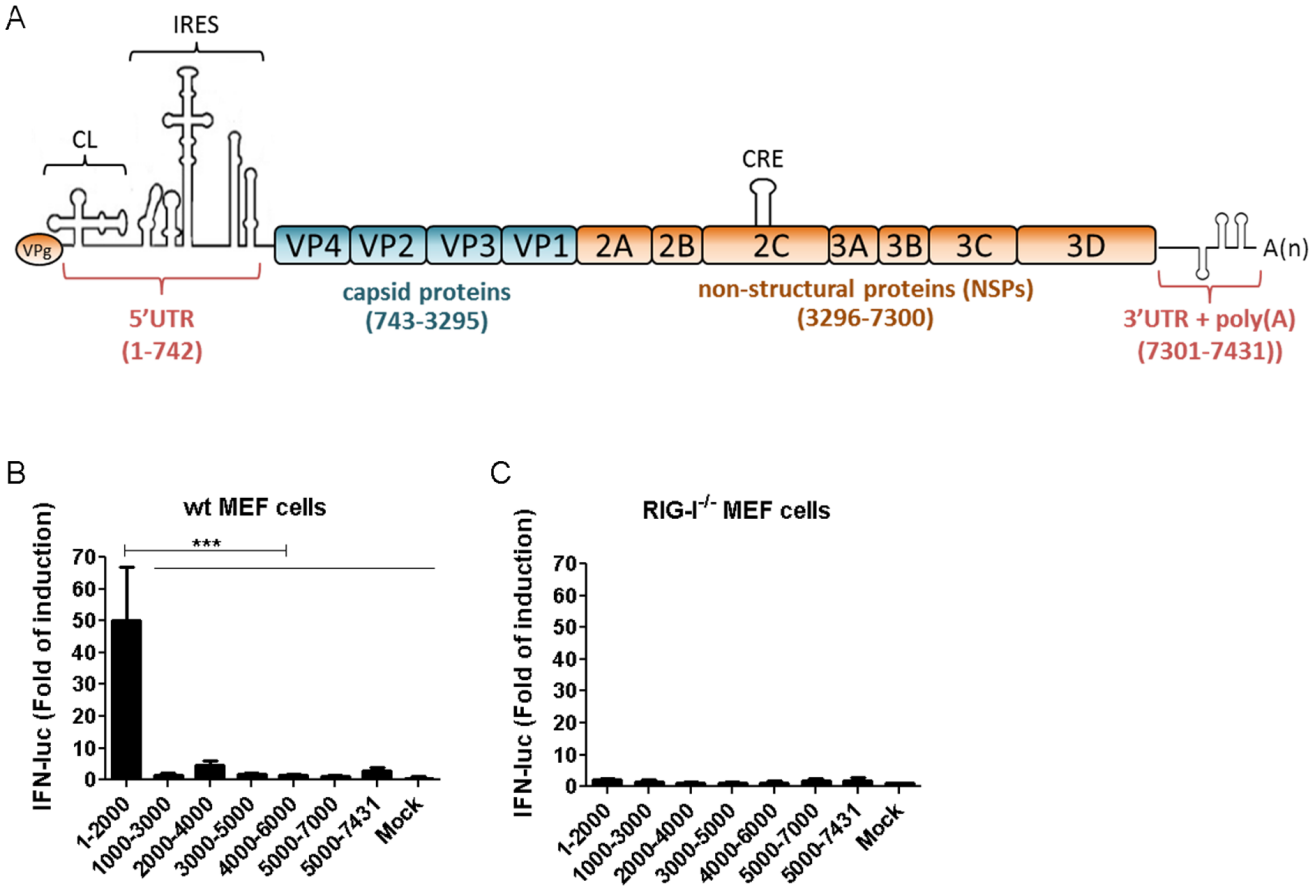
In vitro transcribed RNAs of the first 2-I. (A) Schematic representation of known RNA structures in CVB3 genomic RNA. CL, cloverleaf. IRES, internal ribosomal entry site. CRE, *cis*-acting RNA element. Numbers refer to nucleotide positions in the viral genomic RNA. (B, C) In vitro transcribed (ivt) RNAs with sequences corresponding to the indicated positions of CVB3 genomic RNA were transfected into WT (B) or RIG-I^−/−^ (C) MEFs. IFN-β luciferase reporter assay was carried out 8 hours post transfection (hr.p.t.). Data presented as mean +/− SD.

Picornavirus genomic RNA contains several structured elements including the cloverleaf structure (CL) at the extreme 5′ terminus, the stem-loop structures within the viral internal ribosomal entry site (IRES), the *cis*-acting RNA element (CRE) and additional stem-loop structures within the 3′ UTR (Fig. 1A). To investigate whether any of these RNA elements in the CVB3 genomic RNA could stimulate RIG-I, we produced *in vitro* transcribed RNAs (ivtRNAs) corresponding to 2 kb fragments of the CVB3 genomic sequence with 1 kb overlaps, and investigated their individual abilities to activate RIG-I by an IFN reporter assay. Quality and concentrations of these transcripts were confirmed by gel electrophoresis prior to transfection (data not shown). Upon transfection in wt mouse embryonic fibroblasts (MEFs), ivtRNA corresponding to nucleotide (nt) 1–2000 of CVB3 genome (CVB3 1–2000) induced significantly higher levels of IFN-β promoter activation than all other segments of the genomic RNA (Fig. 1B). When RNA transfection was carried out in RIG-I^−/−^ MEFs none of the RNA fragments induced any significant IFN-β activation (Fig. 1C), demonstrating that the responses observed in Fig. 1B were indeed dependent on RIG-I.

**Figure 2:**
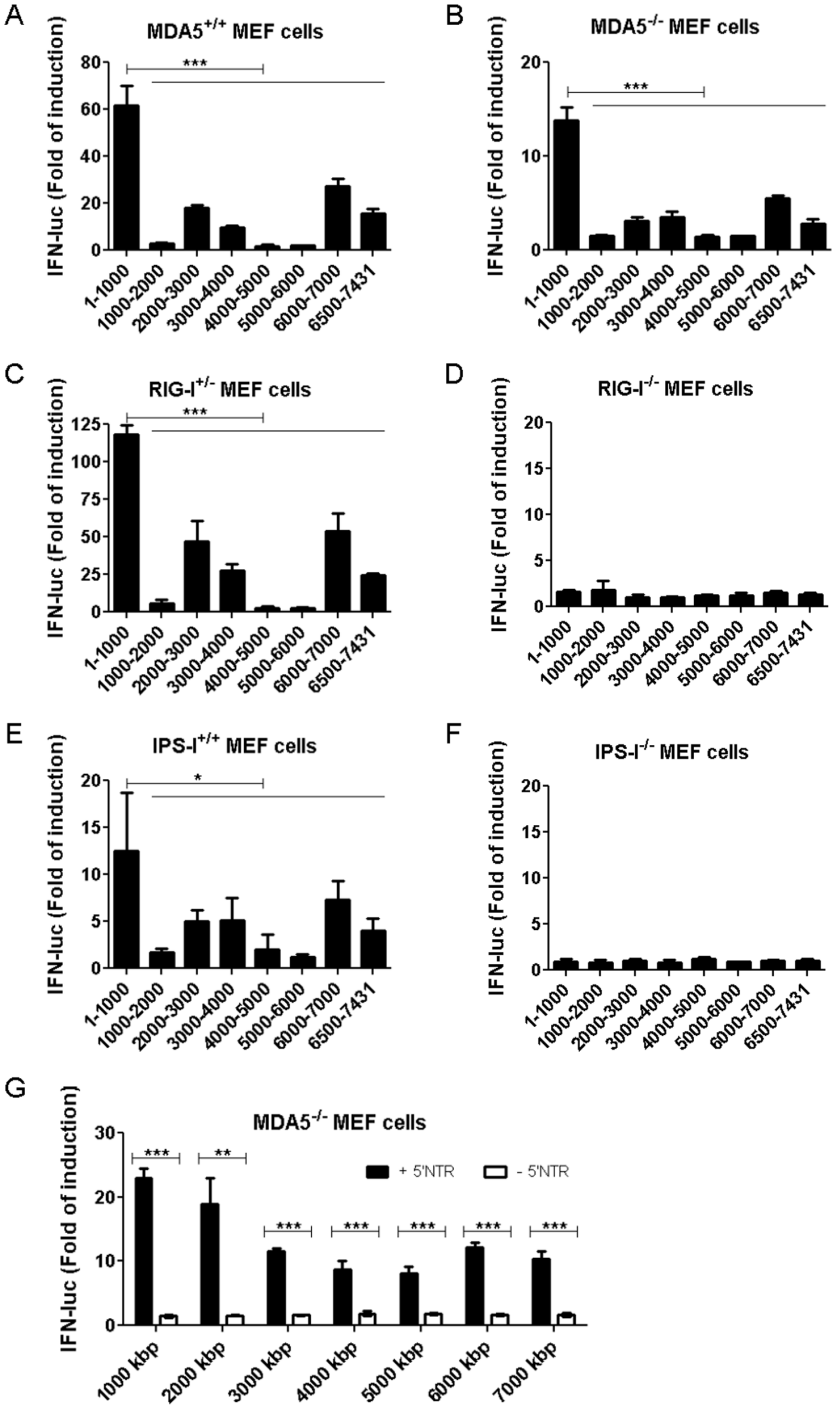
ivtRNA of the first 1-I. ivtRNAs with sequences corresponding to the indicated positions of CVB3 genomic RNA were transfected into MEFs from MDA5^−/−^ (B), RIG-I^−/−^ (D), IPS-I^−/−^ (F)mice or their WT litter controls (A, C, E). IFN-β luciferase reporter assay was carried out 8 hr.p.t.. Data presented as mean +/− standard deviation (SD). (G) ivtRNAs with sequences corresponding to the indicated positions of CVB3 genomic RNA were transfected into WT or RIG-I^−/−^ MEFs. IFN-β luciferase reporter assay was carried out 8 hr.p.t.. Data presented as mean +/− SD.

To further refine the RNA element that activates RIG-I, shorter, 1 kb, 5′ppp-containing RNA fragments of CVB3 genomic sequence were generated, and their RIG-I-stimulatory activities were measured upon transfection into MEF cells. To demonstrate the contribution of cytoplasmic RNA sensors RIG-I and MDA5, transfections were performed in wt MEFs, as well as RIG-I^−/−^, MDA5^−/−^ and IPS-1^−/−^ MEFs. All wt MEFs showed similar patterns of IFN-β induction, and the first 1 kb (CVB3 1–1000) was the most potent IFN-β inducer amongst all fragments tested (Fig. 2A, 2C and 2E). The positive signals were completely abolished in RIG-I^−/−^ and IPS-1^−/−^ MEFs (Figs. 2D, 2F) but not in MDA5^−/−^ MEFs (Fig. 2B), indicating that RNA activity was mediated by the RIG-I/IPS-1 signaling pathway.

To confirm that the first 1 kb of CVB3 sequence is truly the RNA element recognized by RIG-I, ivtRNAs of different lengths were produced, with or without the first 1000 nts of the genomic sequence and assayed their abilities to activate RIG-I. Upon transfection of all fragments including the first 1 kb and spanning 1–7 kb in total, we observed efficient activation of the IFN-β promoter (Fig. 2G). However, when RNAs of the same length, but lacking the first 1 kb sequence, were transfected into cells, only background levels of IFN-β promoter activity were observed (Fig. 2G). These results clearly demonstrate the importance of the first 1 kb of CVB3 genomic sequence in activating RIG-I.

### 5′ppp-containing CVB3 cloverleaf (CL) structure is a potent RIG-I ligand

**Figure 3:**
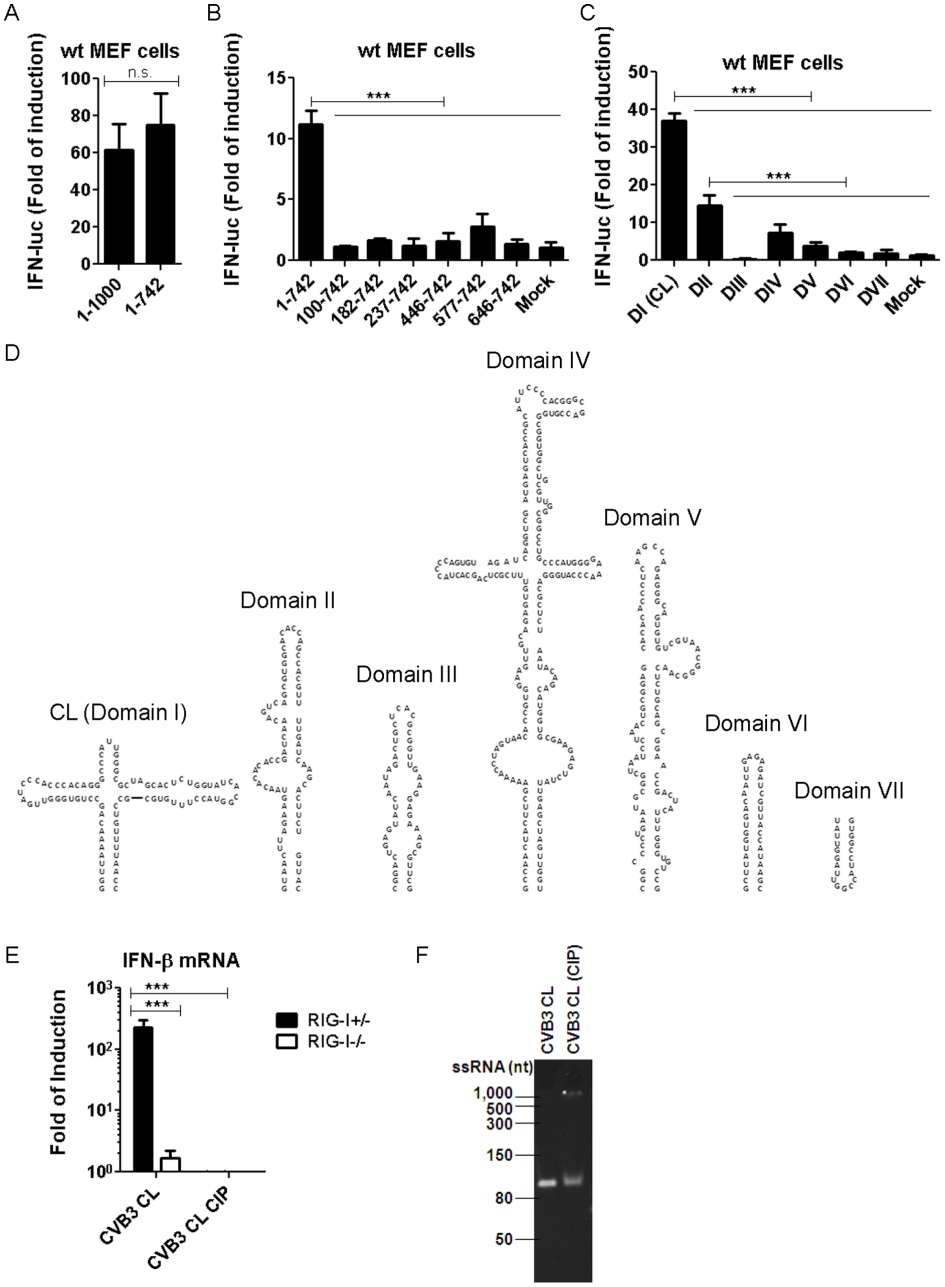
CVB3 Cloverleaf is a potent RIG-I agonist and protects against subsequent EV71 infection. (A, B) ivtRNAs with sequences corresponding to the indicated positions of CVB3 genomic RNA were transfected into WT MEFs. IFN-β luciferase reporter assay was carried out 8 hr.p.t.. Data presented as mean +/− SD. (C) ivtRNAs of individual domains in CVB3 5′ UTR were transfected into WT MEFs. IFN-β luciferase reporter assay was carried out 8 hr.p.t.. Data presented as mean +/− SD. (D) Schematic representation of RNA ligands used in C. (E) CVB3 CL was mock-treated or treated with calf intestinal phosphatase (CIP), and transfected into RIG-I^+/−^ or RIG-I^−/−^ MEFs. IFN-β mRNA levels were determined at 8 hr.p.t.. Data presented as mean +/− SD. (F) RNAs used in E were analyzed on an 8 M Urea/8% Acrylamide gel, and visualized with SYBR Gold staining. RNA fragments were transfected at equimolar amounts in each experiment.

In addition to the 5′ppp moiety present in all RNA fragments tested so far, RIG-I also requires double-stranded regions for recognition [3]. The first 1 kb of CVB3 genome contains the large (nt 1–742) and highly structured 5′ UTR (Fig. 1A), comprised of several stem-loop structures (further referred to as Domains I through VII), which were based on M-fold predictions as well as extensive RNA probing experiments (reviewed in [22]). These RNA elements are essential for viral translation and RNA replication, and therefore, are structurally highly stable and conserved among related viruses [23]. To test our hypothesis that the 5′ UTR is the main RIG-I-stimulating RNA sequence within the first 1 kb of CVB3 sequence, we generated an ivtRNA containing only nt 1–742 (5′ UTR) of CVB3 and compared its ability to activate RIG-I with that of nt 1–1000. Equimolar amounts of RNAs were transfected because RIG-I is known to bind to the termini of RNA ligands (*i.e.* in a 1∶1 RIG-I:RNA molecular ratio). Under these conditions, nt 1–742 induced IFN-β promoter activation to the same extent as nt 1–1000 (Fig. 3A), suggesting that the 5′ UTR sequence is the main RNA element that is recognized by RIG-I. This recognition is dependent on the 5′ppp since treatment of 5′ UTR with polyphosphatase, which yields 5′ monophosphate, or calf intestinal phosphatase, which yields 5′ OH group, completely abolished IFN-β induction (data not shown).

To further refine the RNA region with the highest potency of RIG-I stimulation, we truncated the 5′ UTR sequence from the 5′ terminus, one stem-loop at a time, transfected equimolar amounts of the resulting RNAs, and measured IFN-β response by reporter assay. Surprisingly, loss of the first 100 nt of the 5′ UTR, which corresponds to the CL structure, almost completely abolished the IFN-stimulatory activity of the RNAs (Fig. 3B), strongly implicating the CL as a potent RIG-I ligand. In line with this observation, when ivtRNAs corresponding to each individual domain within the 5′ UTR (Fig. 3D) were transfected into cells, CL induced the highest level of IFN-β activation (Fig. 3C).

Having established that the CL is a potent IFN-β inducer, we set out to confirm that this response is truly dependent on RIG-I. To this end we transfected RIG-I^+/−^ and RIG-I^−/−^ MEFs with CL and determined IFN-β mRNA upregulation levels by RT-qPCR. In addition, we also treated the CL RNA with Calf Intestinal Phosphatase (CIP), which digests the 5′ppp moiety that is required for ssRNAs to activate RIG-I, and studied the resulting RNA to activation IFN-β response in RIG-I^+/−^ and RIG-I^−/−^ MEFs. As shown in Fig. 3E, the CL RNA induced high levels of IFN-β activation in RIG-I^+/−^, but not RIG-I^−/−^ MEFs, and CIP treatment completely abolished the observed IFN-β response. To assess whether CL-induced RIG-I activation is due to copy-back species, which have been described as byproducts of T7 polymerase-produced RNAs [14], [24], we analyzed this RNA (before and after phosphatase treatment) on a denaturing gel. As shown in Fig. 3F, only a single RNA species was present in our RNA preparations, which corresponded to the expected size of CL. This result also excludes that the lack of RIG-I activation by the phosphatase-treated CL is due to degradation of this RNA during treatment. Together, these results clearly identify CL as a potent RIG-I ligand.

### CVB3 CL induces potent IFN-α/β and ISG induction

**Figure 4:**
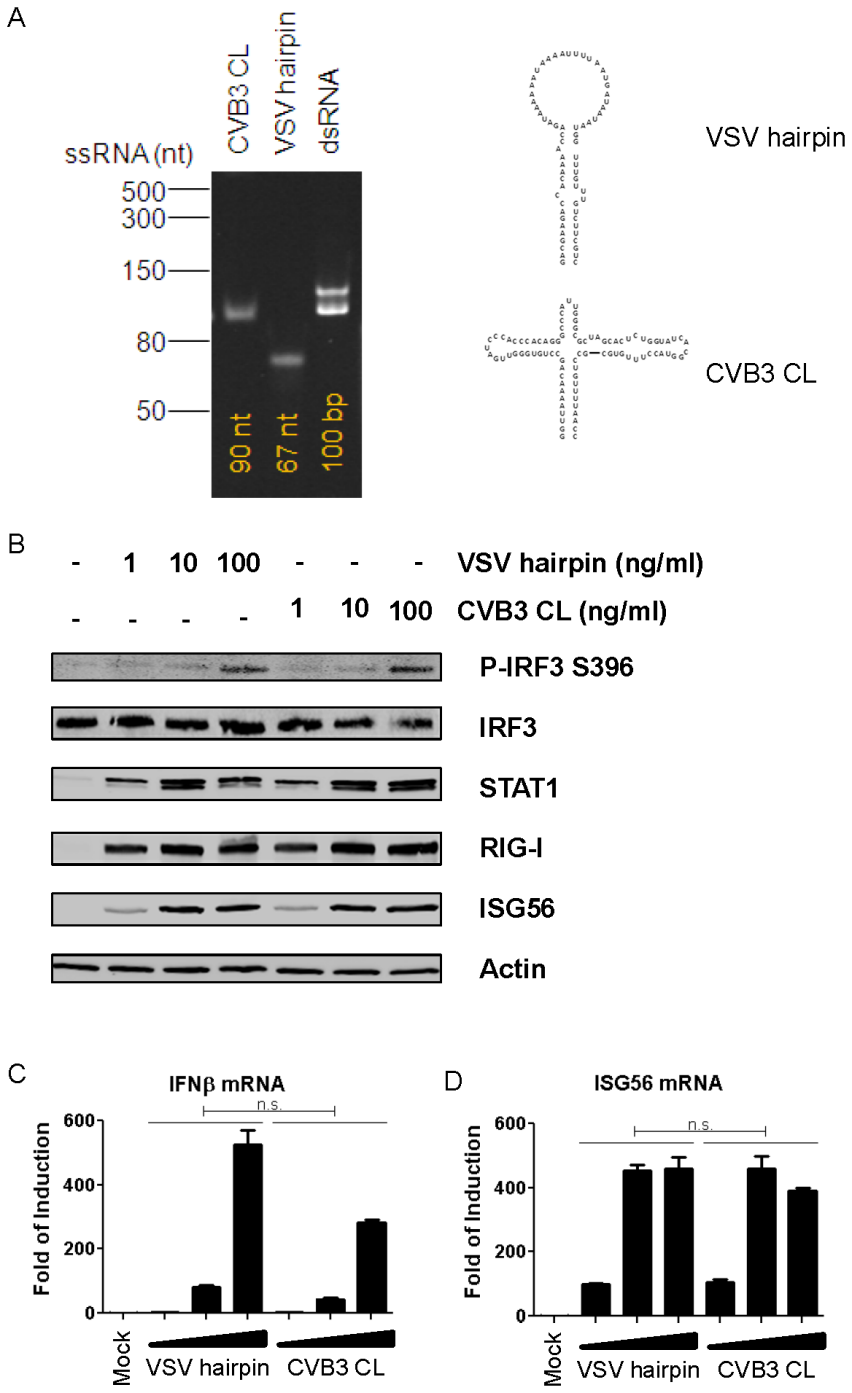
CVB3 CL activates IFN and ISG as efficiently as published ligand. (A) CVB3 CL (with and without CIP treatment), VSV hairpin and a short dsRNA control of 100 bp) were analyzed on an 8 M Urea/8% Acrylamide gel, and visualized with SYBR Gold staining. (B) A549 cells were transfected with indicated amounts of VSV short hairpin RNA or CVB3 CL RNA. Cells were lysed at 24 hr.p.t. and subjected SDS-PAGE analysis followed by immunoblotting using antibodies against the indicated proteins. (C, D) A549 cells were transfected with 0.1, 1 or 10 ng/ml of VSV short hairpin RNA or CVB3 CL RNA and incubated for 24 hrs. Total RNA was isolated and subjected to qRT-PCR analysis using primers specific for IFNβ or ISG56 mRNAs. Data presented as mean +/− SD.

We next sought to compare the antiviral activity of the CVB3 CL to that of the recently described short hairpin RNA derived from VSV UTR sequences (Fig. 4A) that was identified as a potent RIG-I agonist [13]. To confirm the lack of copy-back species in the VSV hairpin preparation we also analyzed this RNA on denaturing gel. As shown in Fig. 4A, both CVB3 CL and the VSV hairpin migrated as single RNA bands of their expected sizes. In comparison, a dsRNA control yielded two bands on denaturing gel. Next, we transfected varying amounts of CL or VSV hairpin RNA into A549 cells, and analyzed the phosphorylation status of IRF3 at Serine 396, a marker of early activation and prerequisite to RIG-I-mediated type I IFN induction. As shown by immunoblotting using an antibody specifically against S396 phosphorylated IRF3, both VSV hairpin and CVB3 CL led to equal levels of IRF3 activation (Fig. 4B). We also assayed expression of several IFN-stimulated genes (ISGs) including STAT1, RIG-I and ISG56 by immunoblotting, and found that the induction profiles of all these proteins were identical upon transfection of equal amounts of VSV hairpin or CVB3 CL (Fig. 4B). Furthermore, profiles of IFN-β (Fig. 4C) as well as ISG56 (Fig. 4G) mRNA induction upon transfection of these two RNA ligands were also similar. We also compared the RIG-I-stimulatory activities of CVB3 CL and VSV hairpin with a 5′ppp-containing dsRNA of comparable size (100 bp), which is also known to activate RIG-I [15], [25]. All three RNA species induced similar levels of RIG-I-mediated IFN-β activation (Fig. S1).

### CVB3 CL induces a broad-spectrum antiviral response

**Figure 5:**
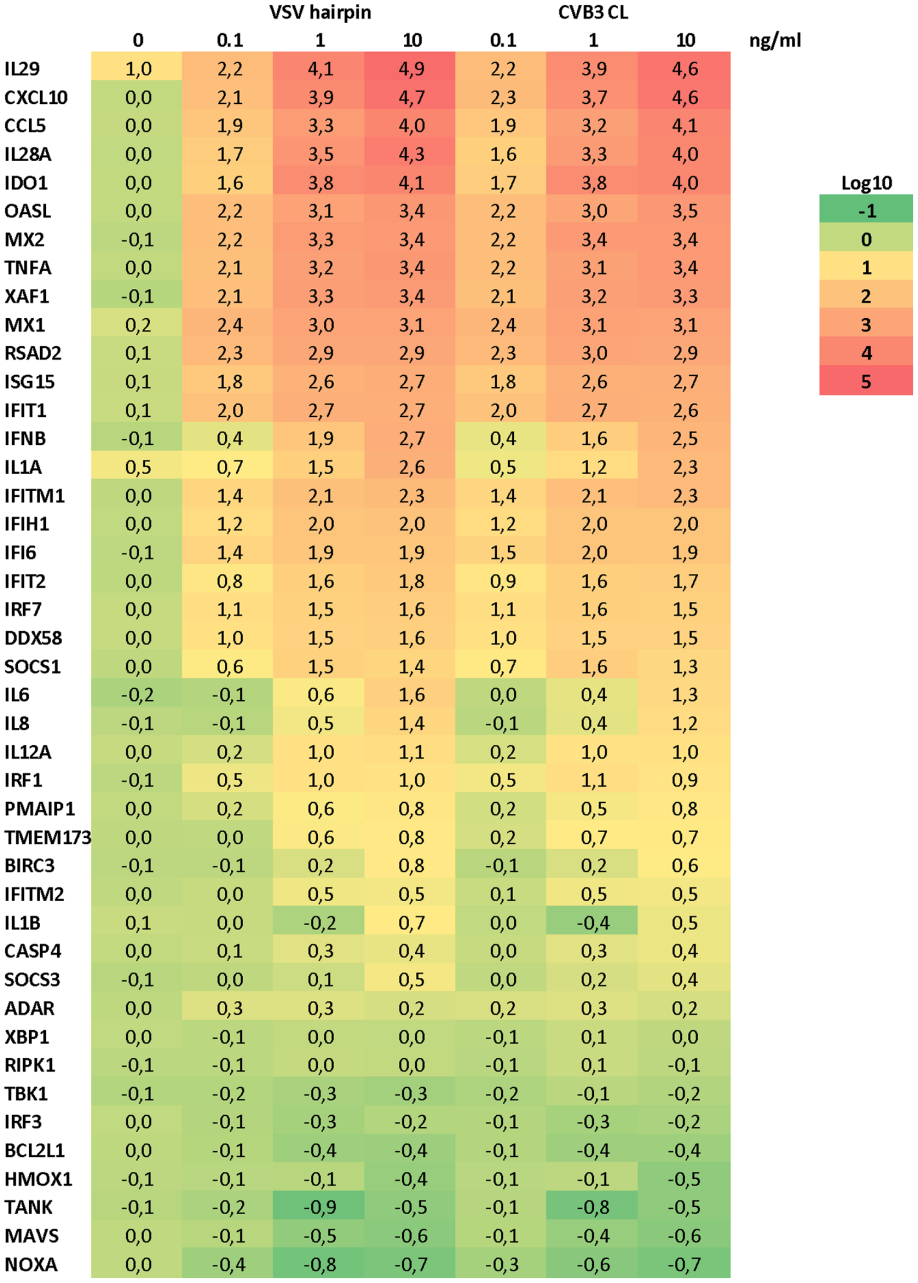
Gene induction profiles of CVB3 CL and VSV hairpin. A549 cells were transfected with indicated concentrations of CVB3 CL or VSV hairpin for 24-PCR analysis using the BioMark system. Data presented as fold of induction in log10 scale as compared to mock-treated sample.

By employing the BioMark large-scale qRT-PCR analysis platform, we also analyzed the cytokine and chemokine induction profile of CVB3 CL as compared to that of VSV hairpin. As shown in Fig. 5, the gene induction profiles by these two RNA ligands were nearly identical for all genes analyzed. In addition to classical ISGs such as CCL5 (RANTES), IFIT1/2, and IFITM1/2, we also observed induction of type III IFNs (IL-28a, IL-28b, and IL-29). In addition, proinflammatory cytokines and chemokines including TNF-α, IL-1α/β and IL-6 were also efficiently unregulated. Two inhibitors of IFN signaling, SOCS1 and 3 were also induced upon transfection of CVB3 CL and VSV hairpin, indicating that the negative feedback regulation of type I IFNs was also activated. These results demonstrate that CVB3 CL activates the RIG-I pathway as potently as VSV hairpin, an established RIG-I ligand with a classical panhandle structure.

### CVB3 CL treatment protects cells against challenge virus infections

**Figure 6:**
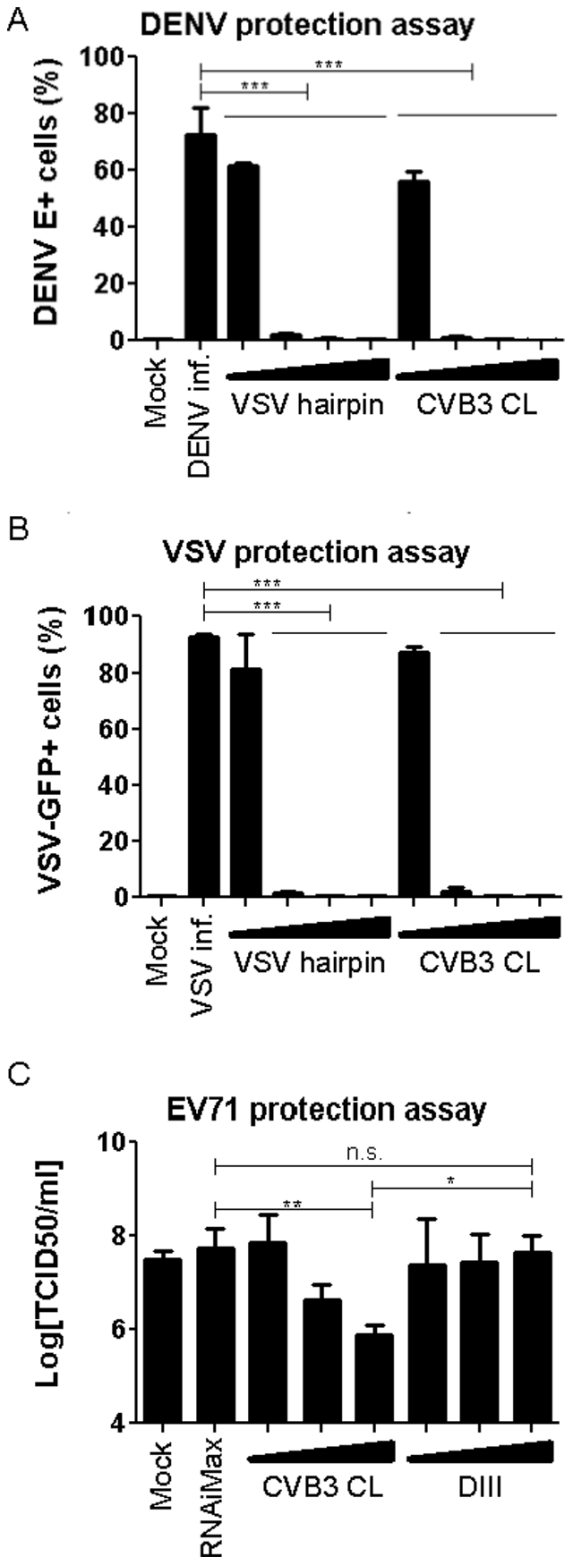
CVB3 CL protects cells against DENV and VSV infections. (A) A549 cells transfected with 0.1, 1, 10 or 100 ng/ml RNA constructs for 18 hrs prior to DENV challenge at MOI of 0.5. Cells were fixed 24 hrs after infection and stained with an antibody against the viral envelop (E) protein. DENV E+ cells were quantified and presented as percentage of the total population. Data plotted as mean +/− SD. (B) A549 cells were transfected with 0.1, 1, 10 or 100 ng/ml RNA constructs for 18 hrs prior to VSV-GFP challenge at MOI of 1. GFP-positive cells were quantified at 24 hrs post infection and presented as percentage of the total population. Data plotted as mean +/− SD. (C) HeLa cells were transfected with 1, 10 or 100 ng cloverleaf (CL) or Domain III (DIII) and incubated for 16 hr. They were then infected with enterovirus (EV) 71 at an MOI of 0.05 for 8 hrs. Virus titers were determined by end point titration on HeLa cells. Data shown as mean +/− SD.

To further confirm the potency of CVB3 CL in triggering antiviral immunity, we performed infections with dengue virus (DENV) and VSV, in cells pre-treated with varying concentrations of CVB3 CL or VSV hairpin. As shown in Fig. 6, both RNAs provided protection against both viruses (Fig. 6A, B) at concentrations of 1 ng/ml and higher. This antiviral effect was not due to cytotoxicity since no significant change in cell viability was observed for either ligand at concentrations of up to 100 ng/ml (data not shown). Together, these data convincingly demonstrate that CVB3 CL is a potent immune stimulator and can effectively induce an antiviral response in transfected cells.

Not all 5′ppp stem-loop structures induced a RIG-I-mediated type I IFN response (Fig. 3C), and we next asked whether the observations with the IFN-β reporter assay were consistent with the antiviral protection. To this end, HeLa cells were transfected with various amounts of CL or Domain III, infected with enterovirus 71 (EV71) 20 h later, and virus titers at 8 hours post infection were determined by end-point titration. In mock-treated as well as lipid alone-treated cells, EV71 infection led to high levels of virus replication (Fig. 6C). Introduction of CL at 100 ng reduced virus titer by approximately 100 fold, and this protective effect was reduced with decreasing amounts of CL transfected (Fig. 6C). Domain III did not protect cells against EV71 infection even at the highest dose used (Fig. 6C), consistent with the earlier observation that this RNA was a poor inducer of IFN-β promoter activity (Fig. 3C).

## Discussion

PRR ligands are currently being investigated as potential antiviral agents. In this study, we examined several small, structured RNAs derived from CVB3 sequence in their RIG-I-stimulatory activities. Although none of these RNA ligands are natural PAMPs during CVB3 infection, the CL structure, when produced in vitro as a 5′ppp-carrying RNA, proved a highly potent RIG-I ligand. Upon transfection, CVB3 CL induces expression of type I IFNs and type III IFNs, as well as many proinflammatory cytokines and chemokines. This ligand protected cells against various viral infections including EV71, DENV and VSV – completely unrelated viruses that exploit different host factors and pathways for replication.

Another RIG-I agonist, a short hairpin derived from VSV genome, was previously characterized as a potent immune stimulator, capable of inducing ISG expression, similar to recombinant type I IFNs, but also pro-inflammatory cytokines such as TNF-α, IL-1α/β, IL-6, and CXCL10 as a result of NF-kB activation. We compared the potency of this ligand and our CVB3 CL in both immune stimulation as well as functional antiviral efficacy. Both ligands induced ISG and cytokine responses to similar extents at the RNA and protein levels. Consistent with this observation, both ligands fully protected cells against DENV and VSV infections at the same concentration. Moreover, the VSV hairpin was shown to protect mice against lethal influenza virus infection [13]. Although not tested in this study, the fact that a large number of cytokine and chemokine genes were induced to nearly identical extents by CVB3 CL and VSV hairpin suggests that CVB3 CL may also elicit protective antiviral immunity *in vivo*. Altogether, our data demonstrate the reliability of utilizing RIG-I pathway activation as a means of immune stimulation.

To date, most established RIG-I ligands, such as the panhandle structure of negative-strand RNA viruses and 5′ppp RNAs derived from Sendai virus and VSV, all adopt a hairpin structure. In comparison, our CVB3 CL adopts a rather unique structure, forming in total 4 short stems, one of which contains a 3-nt bulge, and 3 small loops. Interestingly, three other 5′ppp-containing RNA molecules tested in this study - Domains III, VI and VII - completely failed to activate RIG-I. These RNAs are all predicted to form hairpin structures, and contain 5′ppp on blunt ends, making them appear typical RIG-I ligands. Although Domains III, VI and VII are smaller (54, 36 and 21 nt, respectively) than CVB3 CL (90 nt), they are still larger than some of the established synthetic RIG-I ligands (reviewed in [3]), and Domain III (54 nt) is, in fact, similar in size as the VSV hairpin tested in this study (67 nt). It has also been reported that the length of dsRNAs can play an important role in determining RIG-I activation strength [15]. However, both Domain IV and VI have comparable or slightly longer double-stranded stems than CVB3 CL, but did not induce as much IFN-β as did CL. Of course, we cannot rule out that some of these predicted structures do not fold into their predicted structures, and therefore, poorly activate RIG-I. It is currently difficult to pinpoint why Domains III, VI and VII were incapable of inducing any detectable RIG-I activation, although these results do suggest that ligand recognition by RIG-I may be more complex than currently understood. These observations imply that identification of potent antiviral RIG-I ligands may require additional fundamental investigation.

Known RIG-I ligands not only include 5′ppp-containing ssRNAs such as CVB3 CL and VSV hairpin, but also short 5′ppp-containing dsRNAs [14], [15], [25]. One may ask whether one type of RNA ligand is generally better than the other. We showed that a 100 bp 5′ppp dsRNA *in vitro* transcript induced similar level of IFN-β response upon transfection as the CVB3 CL and the VSV hairpin RNAs, suggesting that both single-stranded and double-stranded ligands can be potent triggers of the RIG-I signaling pathway. Also conceptually, it may not be particularly beneficial to use single-stranded or double-stranded RIG-I ligands to activation this pathway. With respect to large scale production of RIG-I ligands (*e.g.* as a vaccine adjuvant or antiviral agent), chemical synthesis of the 5′ppp moiety is currently challenging. In this regard, dsRNA ligands may be favorable since it has been suggested 5′ppp is not essential for RIG-I activation by dsRNAs longer than 200 bp [15]. However, producing long dsRNAs in a unified form in large quantities may also pose a technical challenge. Research on exploiting RIG-I ligands as a means of clinical intervention (as vaccine adjuvants or antiviral agents) is still at its infancy. We know little about the *in vivo* safety profile, delivery efficiency and potency of single-stranded or double-stranded RIG-I ligands. Having a large repertoire of various RIG-I-activating RNA molecules (as well as those that fail to do so) may be very important to make better-informed decisions in the future.

Recombinant pegylated IFNs has been used as an antiviral therapy against hepatitis B and C virus infections with tremendous success, but with the price of prolonged severe side effects. Therefore, efforts have been focused on the use of ligands of TLRs and RLRs as inducers of antiviral immunity, because these agonists likely mimic the earliest immune recognition events of a natural antiviral response and induce a more extensive and balanced immune activation. Because of the ubiquitous expression profile of RIG-I, RNA agonists would be able to stimulate virtually any cell type, including those at the site of infection. The identification of a novel RIG-I agonist comprising the CVB3 cloverleaf will contribute to the development of RIG-I-based antiviral agents. The identification of RNA ligands that possess all the characteristics required for RIG-I induction - but fail to do so – further demonstrates that there is much to learn about the stimulation of the innate immune response by natural RNA ligands.

## Materials and Methods

### *In vitro* synthesis of VSV hairpin and CVB3 ivtRNAs

The sequence of 5′pppVSV hairpin RNA was derived from the 5′ and 3′ UTRs of the VSV genome as previously described (Schlee 2009 Immunity) and *in vitro* transcribed RNA was prepared as previously described (Goulet 2013 PLoS Pathogens). CVB3 ivtRNAs were produced by T7-driven *in vitro* transcription using PCR products amplified the CVB3 infectious clone p53CB3/T7 [26]. 5′pppRNAs were purified using Qiagen miRNA Mini Kit (Qiagen) or by precipitation. Integrity and concentration of purified RNA ligands were controlled by agarose gel electrophoresis prior to each transfection experiment.

### Cells culture and transfection

MEFs [27]–[29] and HeLa [21] cells were maintained in DMEM supplemented with 10% FCS and 100 U/ml penicillin-streptomycin, in a humidified incubator in the presence of 5% CO_2._ A549 [30] cells were grown in F12K medium (ATCC) supplemented with 10% FBS and antibiotics. Lipofectamine RNAi Max (Invitrogen) was used for transfection of 5′pppRNAs according to the manufacturer′s recommendations.

### Viruses

Enterovirus 71 (strain BrCr) was produced on HeLa R19 cells and titers determined by cytopathic effect-based end-point titration. DENV (serotype 2 strain New Guinea C) was produced on C6/36 cells and purified by ultracentrifugation through a 20% sucrose cushion. DENV titers were determined by FACS upon E protein staining on Vero cells (ATCC). VSV-GFP bearing the methionine 51 deletion in the matrix protein was kindly provided by J. Bell (Ottawa Health Research Institute, CA). Virus stocks were grown on Vero cells, concentrated from cell-free supernatants by centrifugation at 15,000 rpm for 90 minutes at 4°C. Virus concentration was determined by plague assay.

#### SDS-PAGE and immunobloting analysis

5′ppp-treated cells were lysed in RIPA buffer (50 mN Tris-Hcl PH 8, 1% sodium deoxycholate, 1% NP-40, 5 mM EDTA, 150 mM NaCl, 0.1% sodium dodecyl sulfate) and cleared by centrifugation at 17,000×g for 15 min at 4°C. Cleared lysates were subjected to SDS-PAGE analysis on 4–20% acrylamide Mini-Protean TGX precast gels (Biorad, Hercules, USA). Proteins were electrophoretically transferred to Immobilon-P^SQ^ PVDF membranes (Millipore, Billerica, USA) and then subjected to immunoblotting using indicated antibodies. Anti-pIRF3 Ser 396 and anti-RIG-I antibodies were purchased from EMD Millipore, anti-IRF3 from IBL, Japan, anti-IFIT1 from Thermo Fisher Scientific, anti pSTAT1 Tyr701 and anti-STAT1 from Cell Signaling, and anti-β-actin from Odyssey.

#### IFN-β luciferase reporter assay

IFN-β luciferase reporter assay was performed as previously described [21]. Briefly, approximately 200,000 cells were transfected with 50 ng pTK-Rluc, which encodes renilla luciferase (Rluc) under the control of a constitutively active promotor, and 250 ngpIFN-β-luc, which codes for firefly luciferase (Fluc) under the control of the full IFN-β promoter. 24 hours later cells were transfected with the indicate amounts of RNA ligands and lysed 8 hrs post transfection (hr.p.t.) in 1× passive lysis buffer (Promega). Luciferase activities were measured using Dual Luciferase System (Promega) according to menufacturer's protocols. Fluc signal was first normalized against Rluc signal, then against mock-transfected samples. Differences were analyzed using student t-tests or one-way ANOVA (Tukey's Multiple Comparison Test). Throughout: *, p< = 0.05; **, p< = 0.01; ***, p< = 0.001.

#### Real-time quantitative PCR

Real-time quantitative PCR (RT-qPCR) was conducted as previously described [21].

#### Cell viability analysis

Cell surface expression of phosphatidylserine was measured using an APC-conjugated annexin V antibody, as recommended by the manufacturer (Biolegend). Briefly, specific annexin V binding was achieved by incubating A549 cells in Annexin-V binding buffer (Becton Dickinson) containing a saturating concentration of APC-annexin V antibody and 7-amino-actinomycin D (7-AAD) (Becton Dickinson) for 15 min in the dark. APC-annexin V and 7-AAD binding to the cells was analyzed by flow cytometry, as described previously using an LSRII flow cytometer and FACS Diva software.

#### Fluidigm BioMark assay

Total RNA and cDNA were prepared as described above. Intron-spanning PCR primers were designed using Roche's Universal Probe Library Assay Design Center (www.universalprobelibrary.com) and obtained from the Integrated DNA Technology company (**Table S1**). cDNA along with the entire pool of primers were pre-amplified for 14 cycles using TaqMan PreAmp Master Mix as recommended by manufacturer's protocol (Applied Biosystems). cDNA was treated with Exonuclease I (New England Biolabs). cDNA samples were prepared with 2× FastStart TaqMan Probe Master (Roche), GE sample loading buffer (Fluidigm) and Taq Polymerase (Invitrogen). Assays were prepared with 2× assay loading reagent (Fluidigm, NY, USA), primers (IDT) and probes (Roche). Samples and assays were loaded in their appropriate inlets on a 48.48 BioMark chip. The chip was run on the Biomark HD System (Fluidigm, San Francisco USA), which enabled quantitative measurement of up to 48 different mRNAs in 48 samples under identical reaction conditions. Runs contained 40 cycles. Raw Ct values were calculated by the real time PCR analysis software (Fluidigm) and software-designated failed reactions were discarded from analysis. All data are presented as a relative quantification with efficiency correction based on the relative expression of target gene versus the geomean of (GAPDH+Actin+β2 microglobulin) as the invariant control. Fold of induction as compared to mock-treated sample was plotted on a log10 scale and used to generate the heat map. Statistical analyses were performed as described above.

## Supporting Information

Figure S1 **RNAs used in** **Fig. 4A** **were transfected into RIG-I^+/−^ or RIG-I^−/−^ MEF cells at equimolar amounts.** Cells were harvested at 8 hrs post transfection. IFN-β mRNA level in total RNA isolates was measured by RT-qPCR.
(DOCX)

Table S1 **List of primers and probes used for high throughput RT-q-PCR.**
(DOCX)
