# Supplementary material for: Coxsackievirus Cloverleaf RNA Containing a 5′ Triphosphate Triggers an Antiviral Response via RIG-I Activation
Source: PLoS One. 2014 Apr 23;9(4):e95927. doi: 10.1371/journal.pone.0095927 (PMC3997492; doi:10.1371/journal.pone.0095927)
Supplement: Figure S1 — RNAs used in Fig. 4A were transfected into RIG-I+/− or RIG-I−/− MEF cells at equimolar amounts. Cells were harvested at 8 hrs post transfection. IFN-β mRNA level in total RNA isolates was measured by RT-qPCR. (DOCX) [file pone.0095927.s001.docx]

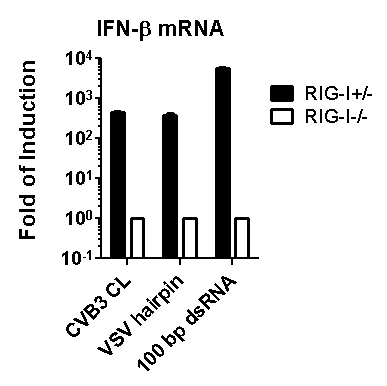
Figure S1

Figure S1. RNAs used in Fig. 4A were transfected into RIG-I^+/-^ or RIG-I^-/-^ MEF cells at equimolar amounts. Cells were harvested at 8 hrs post transfection. IFN-β mRNA level in total RNA isolates was measured by RT-qPCR.
