# Supplementary material for: Coxsackievirus Cloverleaf RNA Containing a 5′ Triphosphate Triggers an Antiviral Response via RIG-I Activation
Source: PLoS One. 2014 Apr 23;9(4):e95927. doi: 10.1371/journal.pone.0095927 (PMC3997492; doi:10.1371/journal.pone.0095927)
Supplement: Table S1 — List of primers and probes used for high throughput RT-q-PCR. (DOCX) [file pone.0095927.s002.docx]

| **TABLE S1.** List of primers and probes used for high throughput RT-q-PCR | | | |
| --- | --- | --- | --- |
| **Gene** |  | **Sequence (5' to 3')** | **Probe** |
| ACTB | F | attggcaatgagcggttc | 11 |
|  | R | tgaaggtagtttcgtggatgc |  |
| ADAR | F | ttcgagaatcccaaacaagg | 39 |
|  | R | ctggattccacagggattgt |  |
| B2M | F | ttctggcctggaggctatc | 42 |
|  | R | tcaggaaatttgactttccattc |  |
| BCL2L1 | F | gctgagttaccggcatcc | 10 |
|  | R | ttctgaagggagagaaagagattc |  |
| BIRC3 | F | gactgggcttgtccttgct | 44 |
|  | R | aagaagtcgttttcctcctttgt |  |
| CASP4 | F | ttgctttctgctcttcaacg | 80 |
|  | R | gtgtgatgaagatagagcccatt |  |
| CCL5 | F | tgcccacatcaaggagtattt | 59 |
|  | R | tttcgggtgacaaagacga |  |
| CXCL10 | F | gaaagcagttagcaaggaaaggt | 34 |
|  | R | gacatatactccatgtagggaagtga |  |
| DENV2 | F | atcctcctatggtacgcacaaa | 5 |
|  | R | ctccagtattattgaagctgctatcc |  |
| DDX58 | F | tgtgggcaatgtcatcaaaa | 6 |
|  | R | gaagcacttgctacctcttgc |  |
| GAPDH | F | agccacatcgctcagacac | 60 |
|  | R | gcccaatacgaccaaatcc |  |
| HMOX-1 | F | ggcagagggtgatagaagagg | 15 |
|  | R | agctcctgcaactcctcaaa |  |
| IDO1 | F | cagcgtctttcagtgctttg | 3 |
|  | R | ggaggaactgagcagcatgt |  |
| IFI6 | F | aaccgtttactcgctgctgt | 40 |
|  | R | gggctccgtcactagacctt |  |
| IFIH1 | F | ggcaccatgggaagtgatt | 20 |
|  | R | gatgatgatattcttcccttcca |  |
| IFIT1 | F | gcctaatttacagcaaccatga | 50 |
|  | R | tcatcaatggataactcccatgt |  |
| IFIT2 | F | atataggtctcttcagcatttattggt | 35 |
|  | R | caaggaattcttattgttctcactca |  |
| IFITM1 | F | cacgcagaaaaccacacttc | 60 |
|  | R | tgttcctccttgtgcatcttc |  |
| IFITM2 | F | tgaaccacattgtgcaaacc | 75 |
|  | R | ctcctccttgagcatctcgt |  |
| IFNA2 | F | aatggccttgacctttgctt | 49 |
|  | R | cacagagcagcttgacttgc |  |
| IFNB1 | F | ctttgctattttcagacaagattca | 20 |
|  | R | gccaggaggttctcaacaat |  |
| IL1A | F | tgacgccctcaatcaaagta | 66 |
|  | R | tgacttataagcacccatgtcaa |  |
| IL1B | F | tacctgtcctgcgtgttgaa | 78 |
|  | R | tctttgggtaatttttgggatct |  |
| IL6 | F | caggagcccagctatgaact | 7 |
|  | R | gaaggcagcaggcaacac |  |
|  |  |  |  |
|  |  |  |  |
| **Gene** |  | **Sequence (5' to 3')** | **Probe** |
| IL8 | F | agacagcagagcacacaagc | 72 |
|  | R | tggttccttccggtggt |  |
| IL12A | F | cactcccaaaacctgctgag | 50 |
|  | R | tctcttcagaagtgcaagggta |  |
| IL28A | F | ccagttccgggcctgtat | 79 |
|  | R | agccaggggactccttttt |  |
| IL29 | F | cctgaggcttctccaggtg | 75 |
|  | R | ccaggaccttcagcgtca |  |
| IRF1 | F | gggctgtcagttgattctgg | 57 |
|  | R | ctatggcacatgcctcaaaa |  |
| IRF3 | F | cttggaagcacggcctac | 18 |
|  | R | cgggaacatatgcaccagt |  |
| IRF7 | F | gcagagccgtacctgtcac | 23 |
|  | R | gcccttgtacatgatggtcac |  |
| ISG15 | F | gcgaactcatctttgccagta | 23 |
|  | R | ccagcatcttcaccgtcag |  |
| MAVS | F | tgcagcaatggtatctgcat | 39 |
|  | R | aaatgattcagcgggagaaa |  |
| MX1 | F | ttcagcacctgatggccta | 79 |
|  | R | aaagggatgtggctggagat |  |
| MX2 | F | cagacctgaccatcattgacc | 9 |
|  | R | tgatgagagccttgatctgc |  |
| NOXA1 | F | gtggatttcctgggcaag | 5 |
|  | R | tcatggttcgctcctggt |  |
| OASL | F | ttgctatgacaacagggagaac | 78 |
|  | R | ctgtcaagtggatgtctcgtg |  |
| PMAIP1 | F | ggagatgcctgggaagaag | 11 |
|  | R | ccaaatctcctgagttgagtagc |  |
| RIPK1 | F | gtgtacaaggggcccaact | 25 |
|  | R | cggctgtgtctcagtctgtt |  |
| RSAD2 | F | tgcttttgcttaaggaagctg | 39 |
|  | R | aggtattctccccggtcttg |  |
| SOCS1 | F | cccctggttgttgtagcag | 36 |
|  | R | gtaggaggtgcgagttcagg |  |
| SOCS3 | F | gacctgaagggaaccatcct | 55 |
|  | R | tgtgttttcggtgactgtcc |  |
| TANK | F | gaggaatagtctacaaaggaagacttg | 80 |
|  | R | actataaaggatggagtaaatgacagg |  |
| TBK1 | F | tgttttgcgagatgtggtg | 72 |
|  | R | cttcccctataacacgcatga |  |
| TMEM173 | F | cgcctcattgcctaccag | 79 |
|  | R | gctgcccacagtaacctctt |  |
| TNFA | F | gacaagcctgtagcccatgt | 79 |
|  | R | tctcagctccacgccatt |  |
| XAF1 | F | cctgccgatcctaaatcaac | 2 |
|  | R | tttccttttgatgaagctaacca |  |
| XBP1 | F | ggagttaagacagcgcttgg | 37 |
|  | R | cactggcctcacttcattcc |  |
